# Compulsory but Unpaid Professional Services

**Published:** 1902-09-20

**Authors:** 


### Compulsory but Unpaid Professional Services.

Among the many kinds of work done gratuitously by the medical profession for the benefit of the public is one which is far more irksome in practice than many people perhaps imagine—namely, the provision of a certificate of the cause of death, which is now by law imposed upon every practitioner who attends a patient in his last illness.

Originally these certificates were given purely as a matter of courtesy, and with the object of improving the accuracy and increasing the value of the returns of the Registrar-General. It always happens, however, that when the medical profession does any good deed for the benefit of the community, the community soon proceeds to render obligatory what was at first done as a matter of grace, and so it was not long before this voluntary and gratuitous certification was made compulsory. No fee was offered, and for years past the ignoble spectacle has been seen of a great and wealthy nation forcing the medical profession to work for it for nothing. The nation possesses a great system of registration. It has a Registrar-General, statistical officers, and hundreds of local registrars, all of them paid and all engaged in reporting, correlating, and tabulating certain facts the importance of which is admitted on every hand. Yet the facts themselves, the professional knowledge on which they are based, the original certificates upon which all this important statistical superstructure is built up, are stolen by Act of Parliament from the brains of the medical profession, every member of which is forced by threat of fine and imprisonment to do this work gratuitously, for the benefit of a nation which is strong enough to take, but mean enough to refuse to pay.

That, however, was not the end of the matter. Ever since the duty of giving these certificates was imposed upon practitioners the coroners have endeavoured, in defiance of the wording of the Act, to induce medical men to withhold the certificates in certain cases, and thus to turn themselves into unpaid coroners' officers; and although they have not succeeded in inducing any large number of medical men to risk a £20 penalty for the sake of currying favour with them, the indirect result of the working of the Act has at least been this—that the registrar reports to the coroner all cases in which the cause given appears to be suspicious, and thus again the gratuitous certificates forced from us as a profession on the plea of obtaining accuracy of registration of disease, are employed to save the police-rate, and we become the unpaid jackals for the coroners and the police. But so tame and voiceless are we under these multitudinous inflictions that now the London County Council proposes a still further "amendment" of the law. It recommends "that a medical practitioner in attendance should be required, before giving a certificate of death, to personally inspect the body, and identify it as the body of the person he has attended;" a very different thing from merely certifying "to the best of his knowledge and belief" the cause of death, for it must be remembered that at present the medical certificate does not certify that the death has even taken place. Who, then, is to pay for these visits to inspect corpses? Is this also to be forced, as another example of gratuitous work, upon this unfortunate profession? Next the doctor is to "include in his certificate a statement pointing to the absence of accident, poison, violence or criminal neglect."

Again, it is proposed that the medical practitioner, instead of handing the certificate to the representatives of the deceased who apply for it, shall be compelled himself to send it to the registrar; and still further, that when the medical practitioner is unable to certify he shall be required to report direct to the coroner. Thus in every way the medical practitioner is to be made the servant of the public, but not a word about payment. We would be the last to urge anything in defence of the present rotten system of death-registration, or of the utter absence of system which not only allows deaths to be registered without certification, but allows bodies to be buried without registration and encourages the occurrence of those gruesome scandals which from time to time arise when an undertaker's premises are found packed with the unburied remains of children who are imagined to be resting in the churchyard. But if the inhabitants of a great and wealthy city require, as they may well require, to have a proper system of registration, and a complete organisation for the discovery of the causes from which its citizens die, let them pay for it, and not adopt the tactics of the highwayman. That medical men bring these things upon themselves by their ever open charity and willingness to do good work unrewarded we admit, but the proposal to compel medical practitioners to do professional work gratuitously for the good of the public is a bit of meanness of which the said public ought to be thoroughly ashamed, and is a piece of extortion the like of which is never attempted in regard to trades and other professions which act, far more than the doctors have ever done, on the principle that the labourer is worthy of hire.

